# A Comparison of Migrant Integration Policies via Mixture of Matrix-Normals

**DOI:** 10.1007/s11205-022-03024-2

**Published:** 2022-10-23

**Authors:** Leonardo Salvatore Alaimo, Francesco Amato, Filomena Maggino, Alfonso Piscitelli, Emiliano Seri

**Affiliations:** 1 Department of Social Sciences and Economics, Sapienza University of Rome, Rome, Italy; 2 Univ Lyon, Univ Lyon 2, ERIC, Lyon, France; 3 Department of Agricultural Sciences, Federico II University of Naples, Naples, Italy; 4 Department of Statistics, Sapienza University of Rome, Rome, Italy

**Keywords:** Mixture of matrix-normals, MIPEX, Model-based classification, Migration policies

## Abstract

In recent decades, there has been a growing interest in comparative studies about migrant integration, assimilation and the evaluation of policies implemented for these purposes. Over the years, the Migrant Integration Policy Index (MIPEX) has become a reference on these topics. This index measures and evaluates the policies of migrants’ integration in 52 countries over time. However, the comparison of very different countries can be difficult and, if not well conducted, can lead to misleading interpretations and evaluations of the results. The aim of this paper is to improve this comparison and facilitate the reading of the considered phenomenon, by applying a Mixture of Matrix-Normals classification model for longitudinal data. Focusing on data for 7 MIPEX dimensions from 2014 to 2019, our analysis identify 5 clusters of countries, facilitating the evaluation and the comparison of the countries within each cluster and between different clusters.

## Introduction

Immigration regulation and immigrants assimilation have been salient political issues in all industrialised countries for many decades, mainly because of their cultural and economic effects (Alesina & Tabellini, 2022). The growing interest in the study of immigration, starting from citizenship and moving more recently to integration, has led to a variety of attempts to quantify immigration policies. Policy indices have become mandatory in the study of immigrant-related policies implemented by different countries. However, the study of these phenomena from a quantitative point of view is rather recent, due to the previous lack or difficulties to access of data (Bjerre et al., 2015). Moreover, quantifying migrant integration is a difficult challenge, linked to its complex nature and lack of uniformity in migration policies of many countries, which are based on multiple criteria.

In this work, we focus on the Migrant Integration Policy Index (MIPEX) (Niessen et al., 2007; Solano & Huddleston, 2020), a complex system of 167 policy indicators across 8 domains of citizenship and integration, combined into a single composite indicator in order to evaluate the migrant integration policies of each considered country over the years. MIPEX has quickly become a solid and useful tool for evaluating and comparing what governments are doing to promote the migrants’ integration in a cross-country setting. Indeed, it informs and engages key policy actors about how to use indicators to improve integration governance and policy effectiveness, with the aim to measure policies that promote integration in both socio-economic and civic terms. Although not without its critics, this index has become a reference for comparative studies on migrant integration over the last decade and its data has been widely used in literature (Hadjar & Backes, 2013; Ruedin, 2015; Rayp et al., 2017; Ingleby et al., 2019). This paper aims to deeply look at how similar, or dissimilar, countries really are and to add new reading perspectives on the MIPEX data, by discovering structures and patterns in the behaviour of the considered countries. The underlying idea is that, given the complex and multidimensional nature of the phenomenon and the differences in socio-economic and civic terms between the examined countries, it can be misleading to compare all of the units with each others. Therefore, the present work aims at improving the analysis, by grouping countries in order to facilitate the comparison and interpretation of the phenomenon. Thus, the research question to which we try to answer:

In order to improve the comparison between the countries regarding their migrant integration policies, is it possible to identify homogeneous groups over time among them, i.e. groups of countries which behave similarly across and within time?

To answer this research question, a Finite Mixture of Matrix-Normals model has been applied to cluster the units, taking into account the longitudinal dimension along 6 years, on the 52 available countries for 7 of the 8 dimensional indicators of the MIPEX. We relied on an unsupervised parametric clustering approach to minimize the risk of arbitrariness^1^ in the choices made and to be able to better evaluate the results.

The paper is structured as follows. Section 2 describes the immigrants integration framework and some works related to migration indicators. Section 3 presents the description of the analysed data and the structure of the MIPEX theoretical framework. In Sect. 4 we present the methodology implemented. Section 5 reports data analysis and the results and Sect. 6 concludes.

## Theoretical Framework and Related works

### Immigrants Integration Framework

Immigration can be generally defined as the set of policies that determine who can enter or exit a country under what conditions, as well as how immigrants are considered once they are settled in a country. Many factors contribute to the migratory flows and stocks (forced or voluntary) to destination countries, which have been extensively addressed in the literature (Dustmann & Preston, 2007; Pedersen et al., 2008; Simpson, 2017). We distinguish short-term migrants (seasonal agricultural workers, students, tourists, or temporary residents) and long-term migrants that include permanent residents, the first step on a path towards the creation of members, namely the citizenship (Goodman, 2019; Solano & Huddleston, 2021). Migration and migrant integration dynamics influence the number and characteristics of migrants entering a country, as well as the integration outcomes (Helbling & Leblang, 2019; Garcés-Mascare nas & Penninx, 2016; Czaika & De Haas, 2013; Massey et al, 1998) At the same time, the receiving society defines all the laws and policies that relate to the selection, admission, integration, settlement, and full membership of migrants in a country (Solano & Huddleston, 2021; Bjerre et al., 2015; Hammar, 1990). Citizenship, migration, and integration policy, albeit in different ways, are distinct policy domains and creates the conditions that support or hinder migrants’ inclusion in the destination society. More attention has been paid to integration policies in recent years, so much so that, in modern countries, they have evolved into very complex legal constructs (Zincone et al., 2011), whereas previously the focus was more on immigrant or assimilation policies. Moreover, as reported in Ramakrishnan (2013), in several countries terms like *assimilation*, *adaptation*, *incorporation* and *integration*, often refer to the same concept and some efforts were needed to provide more conceptual clarity, especially in finding unambiguous definitions of fundamental concepts on the matter. Castles and Davidson (2000) highlight that countries have three main policy options with respect to managing social diversity. The first option is *exclusion*. Although this model is not considered legitimate by humanitarian standards and formally not accepted, it should be noted that it is still predominant in large areas of the world. The second option is *assimilation*. According to this policy model, immigrants should be granted full citizenship: the immigrants’ distinct culture is seen as in transition and it is expected that they fully adopt the national culture and generally accepted social norms. The third option is *integration*, with respect which policy makers are aware that immigrants do not abandon their distinct culture immediately and, therefore, their cultural identity can be considered an opportunity. Legal integration, intended as an immigrant’s legal status, residence rights, citizenship, and equal access to rights, goods, services, and resources, receives wide expert acceptance as the first step in promoting societal integration. It is considered a key determinant (Penninx & Martiniello, 2004) and can hardly be overestimated as either “a firm base” for societal integration or a “clear signal” committing public authorities to an inclusive agenda (Groenendijk et al., 1998). These differences are strictly linked to the complex nature of immigration policies, which involve different political, social and economical spheres that are interconnected with each other. As explained in Niessen and Huddleston (2009), integration is developed by policymakers in conjunction with their policies on social inclusion/cohesion, employment, demography, competitiveness. It follows that immigrant integration is only one part of the broader good governance framework. In recent years, various studies have tried to develop this framework and quantitative indices of immigration policies have been proposed. These indices play a central role in the study of immigrant-related policies, starting with citizenship and moving to immigration and integration (Goodman, 2019, 2015; Helbling, 2013). The next sub-section, although not exhaustively, present some of the most used immigrant-related policy indexes, highlighting how over time they assume greater specificity in relation to integration policies.

### Immigration Policies Indexes: A Literature Review

The policy indices reflect the tendency in social sciences to reduce the complexity of socio-economic phenomena, allowing comparisons across countries and times (Rainer & Marc, 2011; Skaaning, 2010). A sample of immigrant-related policy indexes will be presented below, providing information on index content, type, scope, and source. All of the indices reported in this paper make important and innovative contributions to the field of comparative immigration policy research. It is not our goal to discuss whether and which indexes are better than others. Each index has different methodological and conceptual assumptions and answers specific research questions. In the migratory field, the first index was proposed by Waldrauch and Hofinger (1997) in a study on citizenship, examining the Legal Obstacles to Integration (LOI). But indexing did not stop at citizenship. Several studies have documented the expansion of indexing from citizenship to integration, assuming more specificity for immigration policies (Goodman, 2019, 2015; Helbling, 2013). The first immigrant-related policy indexes proposed, do not differentiate between immigration and integration policy domains. An exception is represented by the index proposed by Boushey and Luedtke (2011), who first consider the distinction between immigration control and immigrant integration measures. This index provides “conceptual clarification to indexing by distinguishing immigration as control policies [that] deal with keeping out “unwanted immigrants” and integration policy as dictat[ing] the transition and settlement of resident immigrants” (Goodman, 2019, p. 579). Recently, an interdisciplinary community of scholars has developed multi-dimensional indices capable of differentiating across types of policies, target groups, and instruments (Goodman, 2019, 2010; Koopmans et al., 2012). We briefly present some of the main ones:

- First released by Banting et al. (2006), the *Multiculturalism Policy Index* (MCP) is a scholarly research project that monitors the evolution of multiculturalism policies in 21 Western democracies. The MCP is designed to provide information about multiculturalism policies in a standardized format that aids comparative research and contributes to the understanding of State-minorities relations. The project provides an index at 3 points in time: 1980, 2000, 2010, and for 3 types of minorities: one index relating to immigrant groups; one relating to historic national minorities; one index relating to indigenous peoples.
- The Migrant Integration Policy Index (MIPEX) (Niessen et al., 2007; Solano & Huddleston, 2020) is a complex system of 167 policy indicators across 8 domains of citizenship and integration combined into a single composite indicator, in order to evaluate the migrant integration policies of each considered country (for details, see Sect. 3).
- Based on the selection of data for 9 countries, between 1999 and 2008, and with the aim of measuring and comparing immigration, asylum, and naturalization policies across countries, the *International Migration Policy and Law Analysis* (IMPALA) database collects comparable data on immigration law and policy across 6 major areas of migration legislation: economic migration, family reunification, humanitarian migration, irregular migration, student migration, and the acquisition and loss of citizenship for migrants resident (Gest et al., 2014; Beine et al., 2016).
- Helbling et al. (2017) presented the *Immigration Policies in Comparison* (IMPIC) project, which proposes a data set that allows to measure immigration regulations.
- *The Canadian Index for Measuring Integration* (CIMI), is an interactive tool that allows for measuring the outcomes of immigrants in Canadian regions. It is a data-driven index that examines 4 dimensions of immigrants’ integration in Canada to assess the gaps between immigrants and the Canadian-born population. The CIMI identifies factors that underline successful immigrants’ integration, assesses changes and trends over time (currently from 1991 to 2020), enables detailed examination of 4 dimensions of integration and provides rankings based on empirical evidence for Canadian geographies.
- The *Immigration Policy Lab* (IPL) (Harder et al., 2018) is a survey-based measure of immigrant integration, to provide scholars with a short instrument that can be implemented across survey modes, with the aim to strike a pragmatic compromise to help generate cumulative knowledge on immigrant integration. The IPL captures 6 dimensions of integration: psychological, economical, political, social, linguistical, and navigational.

With the proliferation of such policy indices, scholars have more refined tools than ever for classifying and comparing policy plans and practices. Immigration and integration policies vary across dimensions, and limiting them to a single dimension reduces the ability to observe variations that could be significant. For this reason, we focused our analysis on MIPEX dimensions instead of the final composite indicator.

## Data

Analyzing a complex phenomenon (Alaimo, 2021b) is often connected to the measuring of some non-directly measurable latent variables (Maggino et al., 2021; Maggino & Alaimo, 2021, 2022). The measurement process in social sciences is associated with the construction of system of indicators. The indicators within a system are interconnected and new properties typical of the system emerge from these interconnections. As it can be easily understood, these kinds of systems are complex systems (Alaimo, 2021a). Therefore, a system of indicators allows the measurement of a complex concept that would not otherwise be measurable by taking into account the indicators individually (Alaimo & Maggino, 2020).

The MIPEX is a system of 167 policy indicators^2^ and it includes 52 countries and collects data from 2007 to 2019, in order to provide a view of integration policies across a broad range of differing environments. The values of each indicator are chosen by experts from each country, by means of a questionnaire. The MIPEX synthetic indicator is constructed by means of an aggregative-compensative approach (Nardo et al., 2005; OECD, 2008; Alaimo & Maggino, 2020). The 167 basic indicators are first aggregated in 58 indicators (for more information, please consult Solano and Huddleston (2020)), which cover the 8 policy areas designed to benchmark current laws and policies against the highest standards through consultations with top scholars and institutions,^3^. The policy areas of integration covered are the following:

- Labour Market Mobility (X1)
- Family Reunion (X2)
- Education (X3)
- Political Participation (X4)
- Long-term Residence (X5)
- Access to Nationality (X6)
- Anti-discrimination (X7)
- Health^4^

For each area, a synthetic measure (dimensional) is calculated as the arithmetic mean of the elementary indicators^5^, i.e. those selected for measuring each policy area. Each dimensional synthetic indicator is bounded between [0, 100]: the higher the value, the better the situation in that policy area.

The method and the approach adopted for the construction of the synthetic index have not been without criticism. Even if it is the most widespread among the aggregation methods for composite indicators construction, the arithmetic mean it has been highly criticized. The main advantage of this method is that it is simple, largely known and gives easy-to-understand results. The main drawback is that it is a full compensative method; consequently, low values in some indicators can be compensated by high values in other ones (OECD, 2008). This assumption is very strong and has a great impact on the results obtained, leading in many cases to an extreme flattening of the differences between the units (Alaimo & Seri, 2021). Despite its success, the aggregative-compensative approach has been deeply criticized as inappropriate and often inconsistent, from both conceptual and methodological point of view (Freudenber, 2003; Maggino, 2017; Fattore, 2017). To address and try to overcome the limitations of this approach, in recent years alternative procedures to synthesis have been developed in the literature (for instance, see: Kerber & Brüggemann, 2015; Kerber, 2017; Alaimo et al, 2021, 2022). However, the purpose of this paper is to improve the analysis of the dimensions of MIPEX in its present form, albeit we suggest a critical read of it. The analysis carried out in the present work uses the listed above dimensions (excluding health), of which we are going to give a brief description in the following sub-sections^6^.

### Labour Market Mobility

Integration of immigrants into the labor market is a process that happens over time and depends on general policies, context, immigrants’ skills and the reason for migration. Labour market mobility policies qualify as only halfway favourable for promoting equal quality employment over the long-term. In most countries, family members and permanent residents can access the labour market and job training, as well as social security and assistance. However, according to Solano and Huddleston (2020), full equality of rights and opportunity in the labour market is still far from being achieved, especially in the public sector.

### Family Reunion

Family reunification policies determine if and when separated families can reunite and settle in their new home. According to Solano and Huddleston (2020), policies are more favourable in traditional destination countries, Northern European countries and new countries of labour migration (e.g. Italy, Portugal and Spain). On the other hand, for family reunification some countries require a high fee to pay and little support (e.g. Austria, Denmark, France, Germany, the Netherlands, Switzerland, UK). Increasingly, countries make exceptions for the highly-skilled and the wealthy, but rarely for the most vulnerable (minors and beneficiaries of international protection).

### Education

Despite being an increasing priority for integration, education is the greatest weakness in the integration policies of many countries. Most immigrant pupils receive little support in finding the right school or class, or in ‘catching up’ with their peers. As described in Solano and Huddleston (2020), Australia, Canada and New Zealand have developed strong targeted education policies through multiculturalism, while the US focuses additional support on vulnerable racial and social groups. In contrast, the education systems of Austria, France, Germany and Luxembourg are less responsive to the needs of their relatively large number of immigrant pupils. New destination countries with small immigrant communities offer inconsistent targeted support (e.g. Japan and Central Europe).

### Political Participation

In most countries, foreign citizens are not enfranchised or regularly informed, consulted or involved in local civil society and public life. Political participation is one of the weakest areas of integration (Solano & Huddleston, 2020). Foreign citizens’ political opportunities differ enormously from one country to another. For instance, in Australia, New Zealand and Western Europe, they enjoy greater voting rights, stronger consultative bodies, more funding for immigrant organisations and greater support from mainstream organisations. With the exception of Korea, immigrants in Asian countries enjoy almost none of these rights unless they (can) naturalise. Despite European norms and promising regional practices, political participation is still almost absent from integration strategies in Bulgaria, Lithuania, Romania and Slovakia.

### Long-term Residence

The security of permanent residence may be a fundamental step on the path to full citizenship and better integration outcomes. Permanent residence is a normal part of the integration process in top-scoring countries in the MIPEX composite indicator, such as Canada, most Latin American countries (Brazil, Chile and Mexico), Nordic countries (Finland and Sweden), and few other European countries (Hungary, Iceland, Slovenia, Ukraine). In contrast, many newcomers are ineligible for permanent residence in China, Denmark, Ireland, Israel, Japan, Switzerland and Turkey. Countries rarely reform their legal routes to permanent residence. The limited major reforms of recent years have been driven by the politicisation of immigration. Brazil, Estonia, Macedonia, Russia, and Turkey have removed previous restrictions, while Austria, Denmark, Korea, Norway, Poland, Ukraine and the US have imposed new ones.

### Access to Nationality

Facilitating access to nationality can significantly increase naturalisation rates and boost integration outcomes. Nationality policies are a major area of weakness in most European and non-European countries (Solano & Huddleston, 2020), especially Austria, Bulgaria, the Baltics, Eastern Europe, and India. By contrast, immigrants have favourable opportunities to become citizens in many countries, e.g., Sweden and the traditional destination countries (Canada, New Zealand and US). Since 2014, nationality policies have become more restrictive in Argentina, Denmark, Greece and Italy, while immigrants’ access to nationality has improved significantly in Brazil and Luxembourg and, to lesser extent, in China, Greece, Latvia, Moldova, Portugal, Spain, Switzerland and Turkey.

### Anti-discrimination

Anti-discrimination laws are becoming increasingly widespread. Victims of discrimination are often too poorly informed or supported to take the first step in the long path to justice, so most do not report their experience to the authorities. Victims are best informed and supported to seek justice in traditional destination countries (Canada, New Zealand and the US) and some EU Member States (Finland, Portugal and Sweden). Since the adoption of EU law in 2000, anti- discrimination has been the greatest and most consistent area of improvement in integration policy across Europe. Over the past 5 years, 7 countries have made positive reforms to discrimination policy (Croatia, Finland, Iceland, Ireland, Luxemburg, Slovenia and Turkey) and more than half of the MIPEX countries now protect against ethnic, racial, religious and nationality discrimination in all areas of public life (Solano & Huddleston, 2020). China, India, Japan, Russia and Switzerland are critically behind schedule on these international trends.

## Methodology

The basic finite mixture model assumes that data are drawn from a density modelled as a convex combination of components each of specified parametric form (Green, 2019). The usage of finite mixture models as clustering procedures comes clear when supposing that the population from which we are sampling is heterogeneous and so there are multiple groups. Model-based clustering refers to the use of statistical models to cluster data, where the (multivariate) observations are assumed to have been generated from a finite mixture of component distributions, each regarded as a cluster, whose specific probability distribution has generated the units belonging to it (Titterington et al., 1985; Hennig et al., 2015). Model-based clustering offers the advantage of clearly stating the assumptions behind the clustering algorithm, and allows the analysis benefit from the inferential framework of statistics to address some of the practical questions arising when performing clustering: determine the number of clusters, detecting and treating outliers, assessing uncertainty (Bouveyron et al., 2019). In our case, we deal with longitudinal data; model-based clustering of such data is far from simple. Indeed, longitudinal data, sometimes referred to as panel data, track the same sample taking measurements at different time occasions. They are very different from time series: in the longitudinal case we observe short sequences of data in correspondence to a large number of individuals or statistical units, whereas in the time series case we observe long sequences of data referred to one or few statistical units (Bartolucci et al., 2012). The ideal way to model these data would be to take into account the temporal evolution and models all the responses at the same time. Thus, the analysis will exhibit typical temporal evolution behaviours, which are the objects that researchers in human and social sciences wish to study.

In this paper, we adopt a clustering approach to longitudinal data that consists of arranging the data in a three-way format and modelling them through a matrix-variate mixture model. This approach offers the advantage of accounting for the overall time-behavior, grouping together the units that have a similar pattern across and within time. While not being new (Basford & McLachlan, 1985), matrix-variate distributions have recently gained attention, and Mixtures of Matrix-Normals (MMN) have been developed and applied both in a frequentist framework (Viroli, 2011a) and within a Bayesian one (Viroli, 2011b). From a frequentist point of view, these models represent a natural extension of the multivariate normal mixtures to account for temporal (or even spatial) dependencies, and have the advantage of being also relatively easy to estimate by means of EM algorithm (a nice short description of the EM application to MMN is provided in Wang and Melnykov (2020)). Very recently, Tomarchio et al. (2022) applied MMN to cluster longitudinal students’ career indicators for Italian universities.

### Mixture of Matrix-Normals

MMN, as introduced in Viroli (2011a), can be a useful tool to cluster time-dependent data. Suppose we observe *N* independent and identically distributed random matrices $Y_1,\dots , Y_N$ of dimension $J \times T$, with *J*-variate vector observations measured repeatedly over *T* time points (i.e. $Y \in {\mathbb {R}}^{J\times T}$), as in a longitudinal study case. Assume that *Y* follows a matrix-normal distribution, $Y \sim \mathcal{MN}\mathcal{}_{(J\times T)}(M,\Phi ,\Omega )$, where $M \in {\mathbb {R}}^{J\times T}$ is the matrix of means, $\Phi \in {\mathbb {R}}^{T \times T}$ is a covariance matrix containing the variances and covariances between the *T* occasions or times and $\Omega \in {\mathbb {R}}^{J \times J}$ is the covariance matrix containing the variance and covariances of the *J* variables. The matrix-normal probability density function (pdf) is:

1 $$\begin{aligned}&f(Y\mid M,\Phi ,\Omega )=\nonumber \\&\quad =(2\pi )^{-\frac{TJ}{2}}\mid \Phi \mid ^{-\frac{J}{2}}\mid \Omega \mid ^{-\frac{T}{2}}\exp \left\{ -\frac{1}{2} \text {tr}[ \Omega ^{-1}(Y-M)\Phi ^{-1}(Y-M)^{\intercal }] \right\} \end{aligned}$$

Being a particular specification of the multivariate normal distribution, the matrix-normal distribution shares the same various properties, like for instance, closure under marginalization, conditioning and linear transformations (Gupta & Nagar, 1999). The pdf of the MMN model is:

2 $$\begin{aligned} f(Y\mid \varvec{\pi },\varvec{\Theta })=\sum _{k=1}^K \pi _k \phi ^{(J \times T)}(Y\mid M_k,\Phi _k,\Omega _k) \end{aligned}$$

where *K* is the number of mixture components, $\varvec{\pi }=\{\pi _k\}_{k=1}^{K}$ is the vector of mixing proportions, subject to constraint $\sum _{k=1}^{K}\pi _k=1$ and $\varvec{\Theta }=\{\Theta _k\}_{k=1}^K$ is the set of component-specific parameters with $\Theta _k=\{M_k,\Phi _k,\Omega _k\}$.

Matrix-variate models suffer from over-parametrization that leads to estimation issues. This issue is addressed in Sarkar et al. (2020) and Zhu et al. (2022), with the aim to explain the data with as few parameters as possible. To do so, the spectral decomposition of the covariance matrix (Banfield & Raftery, 1993; Celeux & Govaert, 1995) is used. The spectral decomposition of the general covariance matrix $\Omega _k$ is given by $\Omega _k=\lambda _k \Gamma _k \Delta _k \Gamma _k^{\intercal }$, where $\lambda _k = \mid \Omega _k \mid ^{1/J}$, $\Gamma _k$ is the matrix consisting of the eigenvectors of $\Omega _k$ and $\Delta _k$ is the diagonal matrix composed by the eigenvalues. From a geometrical interpretation point of view, $\lambda _k$ mirrors the volume of the *k*-th mixture component, $\Gamma _k$ the orientation and $\Delta _k$ the shape. In MMN, there are two covariance matrices, one measuring covariance in time and one among variables. For identifiability issues of the model, the determinant of the time-covariance matrix must be restricted to be $\mid \Phi _k \mid = 1$, hence imposing *K* restrictions and making $\lambda _k=1$ for the matrix $\Phi _k$. Moreover, two kinds of mean matrices *M* are considered: a general (no constraints) and an additive one. An additive matrix $M_k$ has the structure $M_k=\alpha _k {\mathbf {1}}_T^{\intercal } + {\mathbf {1}}_J\beta _k^{\intercal }$, where ${\mathbf {1}}_{T}$ represents a *T*-dimensional vector of 1s, $\alpha _k$ is the *J*-dimensional mean vector for the variables (row-wise) and $\beta _k$ is the *T*-dimensional mean vector across time (column-wise). This structure gives rise to identifiability issues, which are resolved by imposing *K* constraints $\beta _{k,T}=0$. Last, as introduced in Mcnicholas and Murphy (2010), the time-covariance matrix can be further decomposed through the modified Cholesky decomposition to parameters interpretable in an Auto-Regressive (AR) fashion. Any or all among volume, shape or orientation can be constrained across mixture components. Following the conventional notation in Bouveyron et al. (2019), for the covariance matrices parameterizations E stands for equal, V denotes variable, I represents identity, configuring different types of constraints that can be imposed. Since $\Omega _k$ can be decomposed in 3 submatrices, and $\Phi _k$ in 2, we have 14 different possible combination for the former and 8 (including AR) for the latter, giving rise to $14 \times 8=112$ different parametrizations. Since the mean matrix $M_k$ can be in turn parametrized with a general or an additive structure, in total we can fit $2 \times 112=224$ differently parametrized models.

## Analysis and Results

Data used are freely downloadable from the Migrant Integration Policy Index website^7^. For sake of brevity, during the analysis and in all the Tables and Figures, we name the indicators using one-word labels or the codes reported in Sect. 3.

**Fig. 1:**
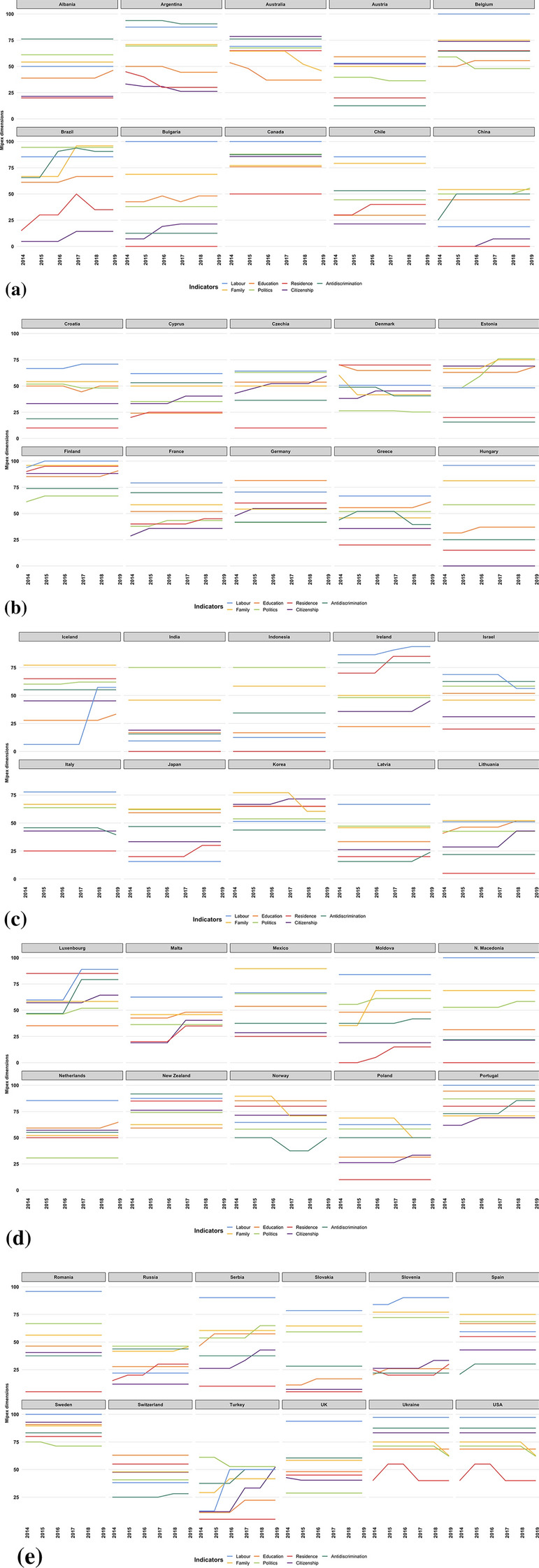
Country trajectories of the 7 MIPEX dimensions. 52 countries; years 2014–2019

The analysis has been carried out by considering 7 MIPEX dimensions explained in Sect. 3. In this paper, we deal with a three-way “time data array” of the type “units $\times$ variables $\times$ times” (D’Urso, 2000) that can be algebraically formalised as follows:

3 $$\begin{aligned} {{\textbf {Y}}}\equiv \ \begin{Bmatrix} y_{ijt}: i=1, \dots , N; \quad j=1,\dots , J; \quad t=1, \dots , T \end{Bmatrix} \end{aligned}$$

where the indices *i*, *j* and *t* stand, respectively, for the units, the quantitative variables and the times. In this paper, $i=1,2,\dots ,52$ indicates the generic country, $j=1,2,\dots ,7$ the generic MIPEX dimensional indicator and $t=204,2015,\dots ,2019$ the generic year; consequently, $y_{ijt}$ represents the determination of the *j*-th indicator in the *i*-th country at the *t*-th year. The first step is to give a geometrical representation of the initial data array ${\mathbf {Y}}$ to obtain information on the form of the data and the relationships between the basic indicators (Pearson, 1956). Figure 1 outlines that the trajectories of most of the indicators appears quite flat, which means that most of the countries does not change much the values of their indicators (and so the related policies) over time. For instance, Canada, India, Indonesia, Mexico and Romania have no improvement or worsening in any indicator during the considered period; while other countries (for instance, Albania, Austria, Hungary, Italy and Latvia) have just a small change in only one of the considered years. We can also observe that in most of the countries (for instance, Belgium, Bulgaria, Canada, and so on) the labour dimension is the one that rank higher; at the same time, the residence dimension rank lower. However, this is not true for most of the Asian countries, where the family and politics dimensions tend to rank higher and the labour dimension lower. The MMN will be used to model together the changes between and within time, grouping together the units which behave similarly across and within time.

The cluster analysis have been performed with the package $\mathsf {MatTransMix}$ (Zhu et al., 2022) of the statistical software R. As usual when performing clustering, the main parameter to set is represented by the number of clusters *K*. Moreover, it is important that the clusters are interpretable (Fraley & Raftery, 1998; Forgy, 1965). Since our dataset is composed by 52 units, we carried out the MMN model for *K* ranging from 1 to 8 and we run the model several times in order to choose the best number of clusters by means of the Bayesian Information Criterion (BIC): the lowest the BIC, the better the model. The selected number of *K* is 5. The best parametrization of the model, as expressed in Sect. 4.1, is A-VEV-VV^8^, which means that the means $M_k$ are better parsimoniously parametrized in additive way, $\Omega _k$ with varying volume, equal shape and varying orientation (in a two components case, it would be ellipsoidal with equal shape) and $\Phi _k$ has both varying shape and orientation.

**Fig. 2:**
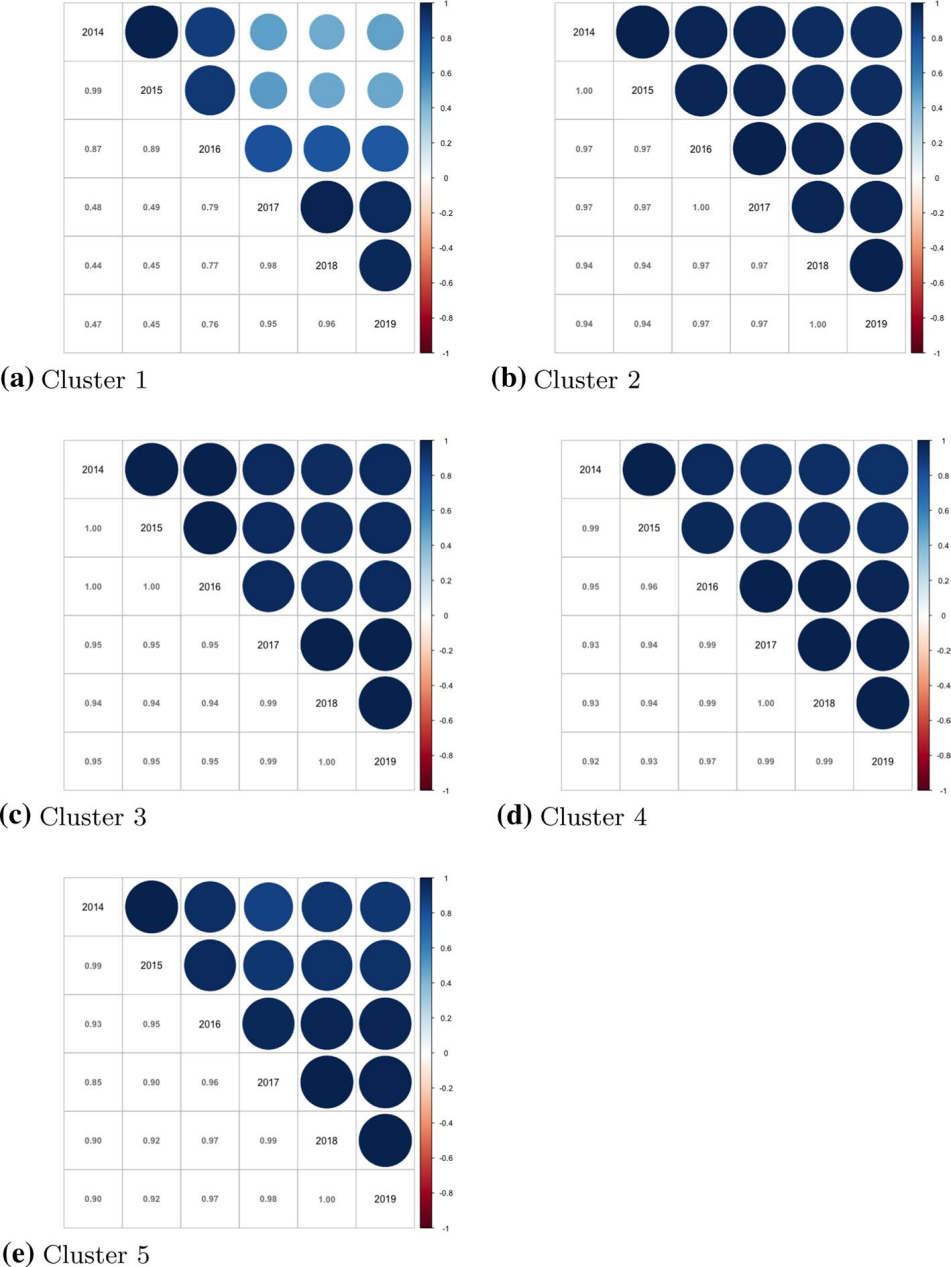
MMN clusters’ corr-plots in time

**Fig. 3:**
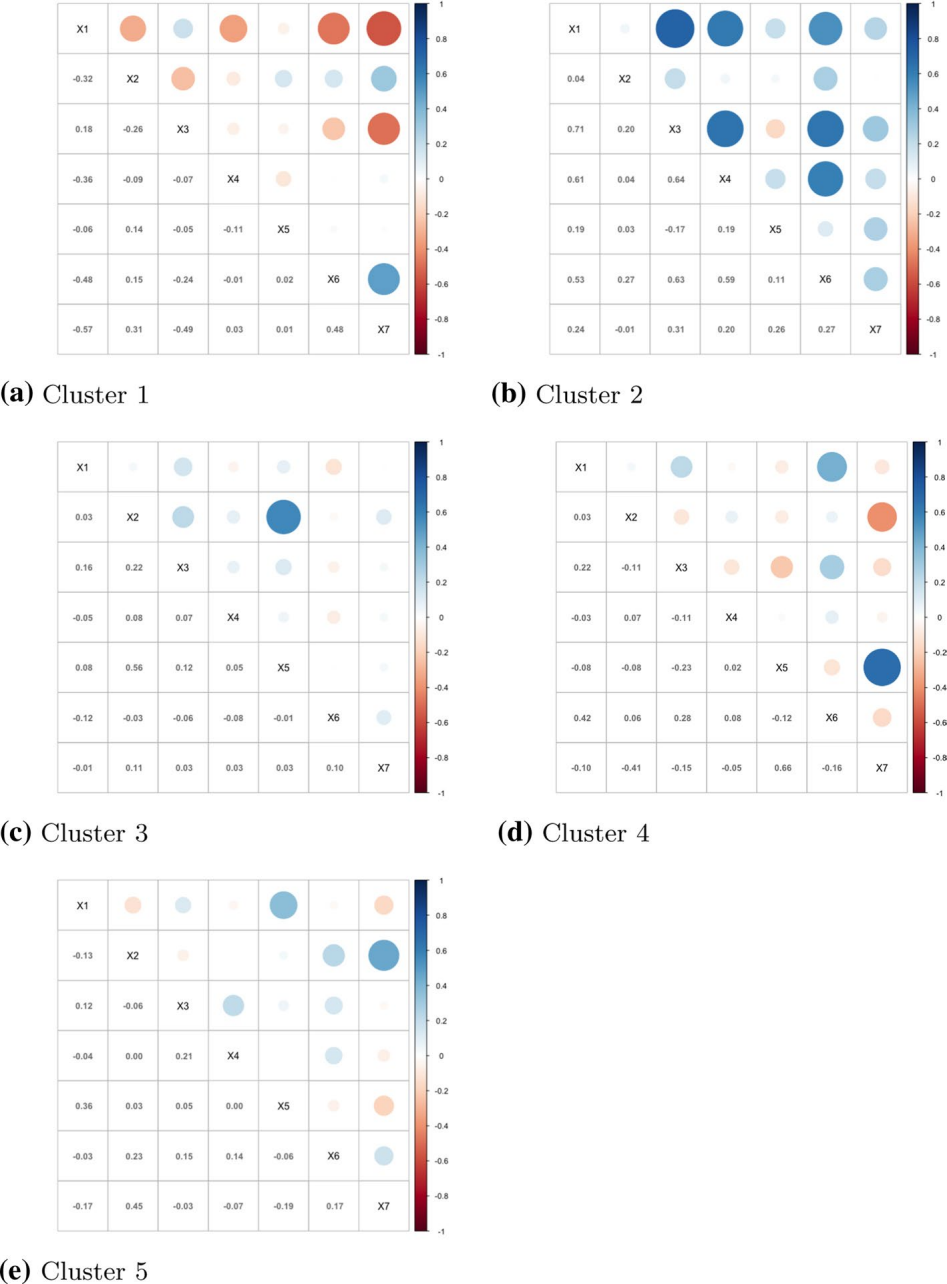
MMN clusters’ corr-plots among indicators. X1 Labour, X2 Family, X3 Education, X4 Politics, X5 Residence, X6 Citizenship, X7 Anti-discrimination

**Fig. 4:**
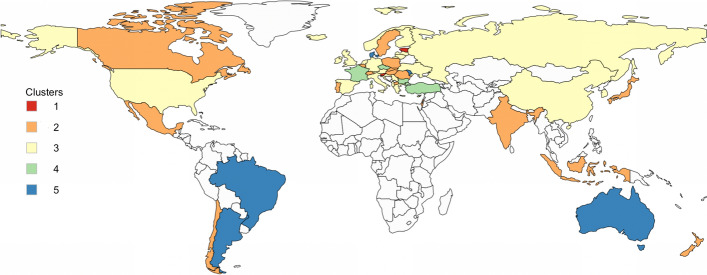
MIPEX dimensional indices: MMN clusters’ composition. 52 countries; years 2014–2019

Because of the matrices $\Phi _k$ and $\Omega _k$, each MMN component models not only the conditional means, but also covariances of the response variables and the covariances among times. This, of course, is visible in the clustering as well, since MMN tends to cluster together not only the units with similar response conditional means, but with conditional covariances among times and variables as well. In this way, each cluster provides a broad profile of units belonging to it. It should be notice that a low correlation in time within cluster means that there have been changes in migration polices in the countries belonging to the cluster; on the other hand, a high correlation in time would signal that little changed. Equally, purified from temporal effect, positive variables correlations mean that the policies’ dimensional scores move homogeneously country-wise within cluster. The values of the correlation in time are reported in Fig. 2, the values of the correlations among variables in Fig. 3 and the countries that belongs to each cluster in Fig. 4. The values of the clusters’ means over time are reported in Table 1.

A description and interpretation of the clustering results is as follow:

- *Cluster 1* Estonia and Slovenia.
  - *Correlation in time* with respect to the other clusters, Cluster 1 is the one with the lowest correlations within time.
  - *Means* this is the cluster with the lowest mean values in the Citizenship strand. With respect to the other clusters, it has low values in the Politics indicator but high values for Family, Residence and Anti-discrimination.
  - *Correlation among indicators* the Labour indicator presents negative correlations with almost all the other indicators except for Family. The correlation is particularly high between the indicators Labour and Anti-discrimination.
  In Cluster 1, we observe relatively low levels of temporal correlation, and this is due to the fact that Estonia has important changes in Family indicator in 2016 and 2017 and Residence in 2017, while Slovenia has important changes in the Anti-discrimination in 2016, in Education in 2018 and Politics in 2019. Cluster 1 is characterized by lower correlations in time between the first 3 years (2014–2016) and the second ones (2017–2019). Moreover, it has negative correlation between Labour Market Mobility and the other dimensions, with the exception of Family Reunion. Countries in this cluster have the lowest score for the Access To Nationality and rank low for Political Participation as well, while ranking high for Family Reunion, Long-term Residence and Anti-discrimination legislation.
- *Cluster 2* Belgium, Canada, Chile, Hungary, India, Indonesia, Israel, Japan, Mexico, New Zealand, North Macedonia, Poland, Portugal, Romania, Slovakia, Sweden, Switzerland.
  - *Correlation in time* Cluster 2 presents high correlation values in time.
  - *Means* with respect to the other clusters, the values of the means of this group are quite low in Politics and Education and high in Family, Residence and Anti-discrimination.
  - *Correlation among indicators* almost all the indicators of this cluster are positively correlated, with particularly high values between Education and Labour, Politics and Labour, Politics and Education, Citizenship and Education and Citizenship and Politics.
  During the analysed period, countries belonging to this cluster did not change much their policies, and they usually rank high in all the areas. The countries of this group tend to have good policies for Residence, Family and Anti-discrimination, but rank low for Education and Politics.
- *Cluster 3* Albania, Austria, China, Croatia, Cyprus, Finland, Germany, Greece, Iceland, Ireland, Italy, Korea, Latvia, Lithuania, Luxembourg, Malta, Netherlands, Norway, Russia, Serbia, Spain, Ukraine, UK, USA.
  - *Correlation in time* Cluster 3 presents the highest correlations in time with respect to the other clusters.
  - *Means* with respect to the other clusters, this group does not present low mean values for any indicator. It presents medium values in Politics, Labour, Family, Education and Citizenship indicators and quite high values in Residence and Anti-discrimination.
  - *Correlation among indicators* almost all the correlations values among indicators are low, with exception for Residence and Family.
  The characteristic of Cluster 3 is its high stability in time, that is the tendency to not make huge changes in the legislation, with some remarkable exceptions such as Iceland in Anti-discrimination in 2018 and Citizenship and Anti-discrimination in Luxembourg in 2017. To this cluster, belongs the countries that reformed less their immigration legislation during the study period. They tend to rank average in most of the policies areas, with the exception of Residence and Anti-discrimination laws, where they tend to rank higher. This group could be seen as the “average” cluster, grouping countries which could be located at the middle of the MIPEX overall rank. This does not mean that any country of this cluster do not present high or low values in any indicator, but that overall, among the indicators the tendency is towards the center. However, low correlation among variables signals that countries do not move homogeneously among the policies areas.
- *Cluster 4* Bulgaria, Czech Republic, France, Turkey.
  - *Correlation in time* it presents high values but they shades with time.
  - *Means* with respect to the other clusters, Cluster 4 have the lowest mean values for Politics and quite low values in Education, Citizenship and Labour. It has high mean values in Anti-discrimination.
  - *Correlation among indicators* it generally presents low correlations with the exception for an high positive value between Anti-discrimination and Residence.
  Cluster 4 is mainly characterised by its relatively low values of Politics in every country, including France. Important positive improvements in Education across time for all the countries mostly explaining the time-correlation behaviour. Despite ranking generally high for Anti-discrimination policies, countries within this cluster tend to rank low for policies in Education, Citizenship and Labour, while scoring average for Residence legislation. Yet, low correlation among variables indicates that the countries do not move homogeneously among the dimensions, with the exception of policies regarding Residence and Anti-discrimination, that have high positive correlation. Countries belonging to this cluster have seen their score moderately changing in time, indicating that some changes in the legislation have happened.
- *Cluster 5* Argentina, Australia, Brazil, Denmark, Moldova.
  - *Correlation in time* it presents high values but they shade faster.
  - *Means* with respect to the other clusters, the values of the means of Cluster 5 are quite low in Education and Politics, medium in Labour and high for the other indicators.
  - *Correlation among indicators* the values of the correlations are generally low.
  Cluster 5 collects countries with smooth evolution, in both positive and negative directions and it generally presents low values in Education (with the exception of Australia). Changes are to be noted in Residence, where all the countries (with the exception of Argentina) see their values change in time (in both directions). Countries belonging to this cluster have high correlation values in time, but they tend to decrease faster with time, meaning that some changes in the policies have been made especially in the last years. Countries of this cluster, are characterized for generally ranking low in policies related to Educational support for foreign pupils and Politics, but high in Family, Residence, Citizenship and Anti-discrimination. However, the low correlation among the dimensions, means that the countries tend not to move homogeneously among them.

Looking at the details of the countries assigned to each cluster, it could be noticed that in the clustering process the algorithm gave more importance to the temporal and variables’ dynamics (captured by $\Phi$ and $\Omega$) than to their overall scores (captured in *M*). The clustering privileged the similarity in trajectory rather than in magnitude. This gives us an idea on how the clustering should be read and explains why countries that one could think are quite different in their policies are in the same cluster.

## Conclusions

This paper has explored immigrant regulation and immigrant assimilation policies, analyzing 7 dimensions of the Migrant Integration Policy Index from the year 2014 to 2019. The need for the analysis carried out came from the statement that when comparing very different countries from each other on social and civil issues, the identification of homogeneous groups of units substantially improves the ease of reading and the interpretation of the results. In this paper, we addressed this issue trough the application of an unsupervised clustering approach for longitudinal data namely MMN. The exploration and visualization of the data show that for the 7 MIPEX dimensions analyzed, the considered countries tend to change little over time. This behaviour led us to rely on an approach as MMN, that accounts simultaneously for the within and between time dependency structures. The identification of groups of countries with similar behaviour over time allows the comparison of clusters with each other and the comparison of the countries within each cluster. Moreover, the correlations in time shows the general trend of each indicator over time in each cluster, and the correlations between variables purified from the time effect underline the behaviour of each indicator in relation to the others within each cluster. This analysis allowed the addition of new levels of interpretation of the migration policies and of several new information about the phenomena. Specifically, the information added helps to better understand which countries have similar legislative attitudes regarding migration policies and which are following similar trends, whether they are virtuous toward integration, static, or toward the marginalization of migrants. For instance, the evidence that Bulgaria and France are both in Cluster 4 highlights that they both have relatively low values for the Politics dimension and they both improved the Citizenship dimension over the considered years.

As future developments of this work, we expect, as the data will be available, to add to the analysis the Health dimension. This would be of particular interest especially during the last years of COVID-19 pandemic. Moreover, if as we expect, there will be changes in the migration policies of many of the countries considered, and, consequently, there will be changes over time in the trajectories of the considered indicators. Moreover, it will be of particular interest to estimate the probabilities to move trough the clusters along the time, through the application of Latent Markov models.

## Appendix

**Table 1:** MMN clusters’ means over time

	2014	2015	2016	2017	2018	2019
*Cluster 1*						
Labour	42.34	42.38	43.62	45.40	45.91	47.02
Family	65.96	66.00	67.24	69.02	69.53	70.64
Education	46.68	46.72	47.96	49.74	50.25	51.36
Politics	19.14	19.18	20.42	22.21	22.72	23.83
Residence	71.85	71.89	73.13	74.92	75.43	76.54
Citizenship	16.64	16.68	17.92	19.71	20.22	21.33
Anti-discrimination	66.12	66.16	67.40	69.19	69.70	70.81
*Cluster 2*						
Labour	48.34	48.34	48.50	48.50	48.56	48.56
Family	64.09	64.09	64.25	64.25	64.31	64.31
Education	39.51	39.51	39.67	39.67	39.73	39.73
Politics	32.52	32.52	32.68	32.68	32.74	32.74
Residence	66.79	66.79	66.95	66.95	67.00	67.00
Citizenship	49.45	49.45	49.60	49.60	49.66	49.66
Anti-discrimination	71.20	71.20	71.36	71.36	71.42	71.42
*Cluster 3*						
Labour	51.65	52.37	52.37	52.95	53.41	53.53
Family	49.99	50.71	50.71	51.29	51.75	51.87
Education	44.42	45.14	45.14	45.71	46.18	46.30
Politics	39.46	40.18	40.18	40.75	41.22	41.34
Residence	58.94	59.65	59.65	60.23	60.70	60.81
Citizenship	49.02	49.74	49.74	50.31	50.78	50.90
Anti-discrimination	65.39	66.11	66.11	66.68	67.15	67.27
*Cluster 4*						
Labour	38.85	39.28	41.76	43.25	43.63	44.56
Family	46.45	46.87	49.35	50.84	51.22	52.16
Education	28.89	29.32	31.80	33.29	33.67	34.60
Politics	11.13	11.56	14.03	15.53	15.91	16.84
Residence	50.61	51.04	53.51	55.01	55.39	56.32
Citizenship	37.59	38.02	40.49	41.99	42.36	43.30
Anti-discrimination	67.18	67.61	70.09	71.58	71.96	72.89
*Cluster 5*						
Labour	52.29	51.65	53.22	54.82	54.03	53.85
Family	62.32	61.69	63.26	64.85	64.06	63.89
Education	34.76	34.13	35.69	37.29	36.50	36.32
Politics	40.98	40.34	41.91	43.51	42.72	42.54
Residence	61.75	61.12	62.69	64.28	63.50	63.32
Citizenship	65.94	65.30	66.87	68.46	67.68	67.50
Anti-discrimination	74.32	73.68	75.25	76.85	76.06	75.88

## Footnotes

1 Subjectivity is an essential element in any measurement process, but its presence does not make the process arbitrary (Alaimo, 2020).

2 A policy indicator is a question relating to a specific policy component of one of the 8 policy areas.

3 The highest standards are drawn from Council of Europe Conventions, European Union Directives and international conventions (for more information see: http://mipex.eu/methodology).

4 This dimensions was excluded from the analysis, because it presents data only available for years 2014 and 2019.

5 The elementary indicators are described in (Solano & Huddleston, 2020).

6 A more extensive explanation is given in (Solano & Huddleston, 2020).

7 https://www.mipex.eu/download-pdf.

8 The total number of estimated parameters is given by $K+(J-1)+KJ(J-1)/2+KT(T-1)/2-K+K(J + T-1) =251$, to be estimated from a total of $J \times T \times N=7 \times 6 \times 52=2184$ observations. For a non parsimoniously parametrized matrix-variate normal mixture the number of parameters would be $K[JT + J(J + 1)/2 + T (T + 1)/2] - 1=454$.
